# Circadian regulation in intervertebral disc degeneration: mechanisms and clinical implications

**DOI:** 10.3389/fmolb.2026.1834561

**Published:** 2026-05-04

**Authors:** Jiarui Zhao, Sibo Wang

**Affiliations:** 1 Department of Bone and joint surgery, Hanzhong Central Hospital, Hanzhong, Shaanxi, China; 2 Department of Spine Surgery, Honghui Hospital, Xi’an Jiaotong University, Xi’an, Shanxi, China

**Keywords:** BMAL1, chronotherapeutic strategies, circadian regulation, intervertebral disc degeneration, mechanical signaling

## Abstract

Circadian rhythms are essential for maintaining intervertebral disc (IDD) homeostasis and regulate cellular metabolism, mechanical responses, and therapeutic efficacy. Core clock genes, especially CLOCK and BMAL1, have central roles in controlling energy metabolism, autophagy, and extracellular matrix production in disc cells. When circadian rhythms are disturbed, the progression of intervertebral disc degeneration (IDD) can be accelerated through increased inflammatory activity and impaired nutrient supply, which is particularly harmful in the avascular environment of the disc. Circadian regulation also influences how disc cells adapt to mechanical stress, thereby helping maintain disc structure and function under both normal and pathological loading conditions. As a result, people who are chronically affected by circadian disruption, such as shift workers or individuals with long-term sleep loss, may have a higher risk of developing IDD. From a therapeutic perspective, strategies based on circadian regulation, including light therapy, time-restricted feeding, and chronotherapy, have shown potential to slow or even reverse degenerative changes by re-establishing synchrony with endogenous biological rhythms. This review summarizes the role of circadian rhythms in IDD homeostasis and IDD progression, and further discusses the clinical significance of circadian-targeted approaches for the prevention and treatment of spinal disorders.

## 1 Introduction

Intervertebral disc degeneration (IDD) is a common pathological process underlying many spinal disorders and is closely associated with chronic low back pain, one of the leading causes of disability worldwide (GBD 2019 Diseases and Injuries Collaborators, 2020). With the progressive aging of the global population, the burden of IDD continues to increase. Imaging studies have shown that more than 90% of individuals over 65 years of age have varying degrees of disc degeneration (Mohd Isa et al., 2022). At the same time, IDD has shown a clear trend toward younger onset. MRI-based studies suggest that approximately 30%–50% of adults aged 30–50 years already present imaging features of intervertebral disc degeneration (Brinjikji et al., 2015). Structural damage caused by IDD can directly reduce spinal stability and lead to persistent pain, nerve compression, and functional impairment (Andersson, 1999). These clinical manifestations not only markedly affect quality of life, but also reduce work capacity and increase healthcare costs (Hoy et al., 2012).

IDD, located between adjacent vertebral bodies, serves as the functional joint of the spine and is composed of three main structures: the inner nucleus pulposus (NP), the surrounding annulus fibrosus (AF), and the upper and lower cartilaginous endplates (CEPs) (Peng et al., 2022). As a shock-absorbing structure, the IDD distributes mechanical loads and buffers axial stress (Mantha et al., 2019). Extracellular matrix imbalance is considered a major contributor to disc degeneration (Takeda et al., 2010). The NP is an avascular and highly hydrated tissue that maintains disc shape and function through regulation of extracellular matrix (ECM) homeostasis (Bian et al., 2017). The AF is a fibrocartilaginous structure located at the disc periphery, where it helps bear spinal loading and reduces pressure on the NP under physiological conditions (Holm et al., 1981). CEP cells, as part of the cartilage endplate, are responsible for nutrient transport and help maintain the avascular microenvironment of the disc (Deer et al., 2019). Current evidence suggests that aging, abnormal mechanical stress, and inflammation are all major factors involved in the pathogenesis of IDD degeneration.

Circadian rhythms are evolutionarily conserved regulatory systems that allow organisms to adapt continuously to environmental changes through coordinated control of multiple physiological processes (Patke et al., 2020). Increasing evidence shows that circadian rhythms play important roles in aging, stress responses, and inflammation (Deyurka et al., 2024). The suprachiasmatic nucleus (SCN) of the hypothalamus is widely regarded as the central pacemaker of the body, coordinating nutrient metabolism, energy balance, redox homeostasis, and behavioral activity through a 24-h oscillatory system (Hastings et al., 2018). Core clock genes, including CLOCK, BMAL1, PER, and CRY, are essential components of circadian regulation. Through a transcription-translation feedback loop (TTFL), the CLOCK/BMAL1 and PER/CRY complexes maintain circadian rhythms at the cellular and tissue levels (Takahashi, 2017) (Table 1).

**TABLE 1:** Circadian regulation of intervertebral disc homeostasis and its therapeutic potential.

Therapeutic strategy	Mechanism	Potential impact	Application scenario	References
Light Therapy	Restores circadian rhythms via the SCN.	Reduces inflammation and slows degeneration	For high-risk groups with disrupted circadian rhythms (shift workers, jet lag)	Touitou et al. (2016), Dibner et al. (2010)
Timed Feeding	Aligns feeding with circadian timing	Enhances autophagy and delays degeneration	For patients at risk of metabolic disorders or needing long-term nutritional intervention	Lai et al. (2023), Ma et al. (2022); Zhang et al. (2019)
Chronotherapy	Times drug delivery to biological rhythms	Improves anti-inflammatory effects and repair	For patients requiring long-term treatment with minimal side effects	Baxter and Ray (2020), Lee et al. (2021b)
Pharmacological Intervention	Regulates clock genes linked to autophagy and inflammation	Enhances autophagy and preserves matrix synthesis	For patients with circadian gene abnormalities or significant inflammation	Yu et al. (2021), Maiese (2017)
Lifestyle Modification	Improves daily habits to maintain circadian balance	Reduces degradation and supports repair.	For preventive interventions in general populations at risk of circadian disruption	Chappuis et al. (2013), Xing et al. (2021), Wang et al. (2021)
Multi-Omics Research and Personalized Treatment	Uses multi-omics to track circadian effects on disc health	Identifies biomarkers for early diagnosis	For patients needing precision treatment and better response to therapies	Guo et al. (2023), Jordan and Lamia (2013), Mei et al. (2022)

SCN, suprachiasmatic nucleus; IL-6, Interleukin 6; TGF-β, transforming growth factor beta; AMPK, AMP-Activated Protein Kinase; mTOR, mechanistic target of rapamycin; BMAL1, Brain and Muscle ARNT-Like 1; REV-ERBα, Nuclear Receptor Subfamily 1 Group D Member 1 (also known as NR1D1).

Recent studies have linked circadian rhythm disruption to the development of many chronic diseases, including metabolic syndrome, cardiovascular disease, and cancer (Scheiermann et al., 2013). In the intervertebral disc, circadian gene expression may influence tissue homeostasis by regulating cellular metabolism, matrix synthesis, and inflammatory responses. For example, inhibition of BMAL1 expression has been associated with metabolic imbalance in disc cells, nucleus pulposus cell dysfunction, and accelerated matrix degradation, while BMAL1-deficient mice develop features of IDD(17). These findings suggest that circadian rhythms may contribute to the maintenance of disc cell function and help delay or mitigate degenerative changes. Therefore, exploring the mechanisms and potential applications of circadian regulation in IDD is of clear scientific and clinical importance.

Current treatments for IDD mainly include conservative management and surgery. However, conservative therapies generally relieve symptoms without stopping or reversing degeneration (Santos et al., 2022). Surgical treatment may be necessary in selected cases, but its long-term efficacy is limited and it carries surgical risks and possible complications (Daly et al., 2016). For example, adjacent segment disease (ASD) may occur after spinal fusion (Aiki et al., 2005). For this reason, there is still an urgent need to develop new strategies that can effectively slow or reverse the progression of IDD.

As an intrinsic regulatory system that affects both systemic physiology and the molecular pathways involved in disc homeostasis, circadian rhythm has emerged as a promising target in IDD management. This review aims to summarize the relationship between circadian rhythms and intervertebral disc health, and to provide new perspectives for future research and clinical practice.

## 2 Molecular mechanisms of circadian rhythms

Circadian rhythms regulate a wide range of physiological processes in the human body and are mainly controlled by the coordinated action of core clock genes, including CLOCK (Circadian Locomotor Output Cycles Kaput), BMAL1 (Brain and Muscle ARNT-Like 1), PER (Period), and CRY (Cryptochrome), among which CLOCK and BMAL1 function as the main positive regulators. The CLOCK gene encodes the CLOCK protein, which forms a heterodimer with BMAL1 and acts as a transcriptional activator complex to drive downstream gene expression, with predominant activity during the night phase (Takahashi, 2017; Partch et al., 2014). This complex binds to E-box elements (CACGTG sequence) in target genes and promotes the expression of PER and CRY. After translation, PER and CRY proteins undergo phosphorylation by CK1ε/δ (casein kinase 1 ε/δ), enter the nucleus, and inhibit CLOCK/BMAL1 activity, thereby suppressing further PER and CRY transcription and forming a stable 24-h cycle (Partch et al., 2014; Qu et al., 2023). Phosphorylation of BMAL1 is an important regulatory step in this process, and CK1ε/δ contributes to this modification (Eide et al., 2005). CRY proteins suppress transcription mainly through direct interaction with the CLOCK/BMAL1 complex, especially via the PAS-B domain of BMAL1, and this process is further influenced by CRY phosphorylation and ubiquitination (Albrecht, 2012). CRY ubiquitination depends on FBXL3 (F-Box and Leucine-Rich Repeat Protein 3) and FBXL21 (F-Box and Leucine-Rich Repeat Protein 21), two ubiquitin ligases that regulate CRY stability and thereby influence circadian precision (Siepka et al., 2007). In addition, the nuclear localization signal (NLS) and nuclear export signal (NES) of PER proteins control their transport between the nucleus and cytoplasm, which is essential for accurate circadian timing (Hastings et al., 2018).

**FIGURE 1:**
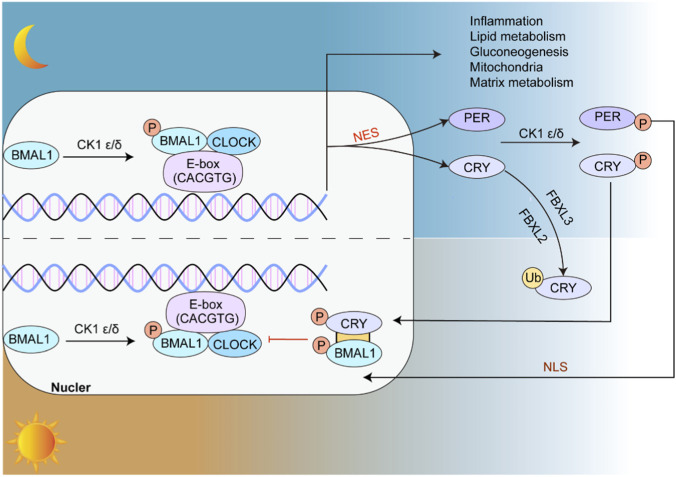
BMAL1 and CLOCK form heterodimers at night and bind to E-box elements to activate downstream genes involved in inflammatory regulation, lipid metabolism, gluconeogenesis, and mitochondrial and matrix metabolism. During the daytime phase, PER and CRY are activated, phosphorylated, and translocate into the nucleus, where they inhibit CLOCK/BMAL1 activity. This transcriptional-translational feedback loop maintains a stable 24-h circadian rhythm.

This interlocked transcription-translation feedback loop (TTFL) not only determines circadian oscillation itself, but also has important effects on cell metabolism and proliferation (Figure 1). For example, the CLOCK/BMAL1 complex can activate downstream genes together with transcription of Rev-erbα (Nr1d1) and Rorα (RORA), both of which are important in lipid metabolism and inflammatory regulation (Preitner et al., 2002; Solt et al., 2012). Some studies have shown that CLOCK deficiency or dysfunction can contribute to metabolic disorders such as obesity and type 2 diabetes (Rudic et al., 2004). In addition, Period2 (Per2) and BMAL1 have been shown to regulate autophagy-related genes, including mTORC1, Atp6v1d (ATPase H + -translocating lysosomal V1 subunit D), ATG4a, ATG4d, Beclin1, Bnip3, Ulk1a, and Ulk1b, thereby affecting autophagic flux (Huang et al., 2016; Wu et al., 2019; Kalfalah et al., 2016).

Circadian rhythms are centrally regulated by the suprachiasmatic nucleus (SCN) in the hypothalamus, while peripheral tissues are controlled by local clock genes expressed in peripheral cells. Representative tissues include the liver, muscle, and bone, where circadian regulation is closely linked to specific physiological functions (Gossan et al., 2013; Dudek et al., 2016). In the liver, circadian rhythms regulate glucose and lipid metabolism as well as the expression of detoxification enzymes.CLOCK/BMAL1 dimerization directly activates the expression of genes such as Pparα and Srebp1, which control several key steps in lipid metabolism (Balsalobre et al., 2000; Zhang et al., 2009). Circadian rhythms also influence gluconeogenesis and glucose output through regulation of genes such as G6pc and Pepck (Dyar et al., 2014). In intestinal epithelial cells, REV-ERBα and RORα help maintain intestinal homeostasis, and this regulatory effect can be strengthened through inhibition of the NF-κB/Nlrp3 inflammatory axis (Wang et al., 2018). In skeletal muscle, circadian rhythms are involved in energy metabolism and muscle repair. The CLOCK/BMAL1 complex affects mitochondrial biogenesis and energy metabolism through regulation of PGC-1α (Halling and Pilegaard, 2020). BMAL1-deficient mice show marked muscle atrophy and reduced strength, supporting an important role of circadian regulation in muscle maintenance (Ray et al., 2020). In addition, the mTOR pathway in muscle is also under circadian control, and BMAL1 can influence protein synthesis and muscle repair by regulating phosphorylation of S6K1 and 4E-BP1 (Lipton et al., 2015). In bone, circadian rhythms participate in bone formation and remodeling. The CLOCK/BMAL1 complex regulates the expression of RUNX2 and Osterix, thereby affecting osteoblast differentiation and function (Dudek et al., 2017). Circadian rhythms in bone also influence the RANKL/OPG system, which helps balance osteoclast activity and bone resorption and is therefore important for maintaining bone mass (Yang et al., 2016) (Table 2).

**TABLE 2:** Circadian molecules and signaling pathways involved in intervertebral disc health.

Aspect	Mechanism	References
Key Circadian Molecules	BMAL1: Regulates autophagy, metabolism, and inflammation, maintaining disc matrix homeostasis under stress	Touitou et al. (2016), Yu et al. (2021), Maiese (2017), Wang and Griffith (2010)
	CLOCK: Partners with BMAL1, influencing circadian rhythm-dependent DNA repair in disc cells	Touitou et al. (2016), Dibner et al. (2010)
	PER1/PER2: Inhibit BMAL1/CLOCK and regulate matrix turnover	Guo et al. (2023), Jordan and Lamia (2013)
	REV-ERBα: Represses BMAL1 and modulates inflammation and metabolism	Yu et al. (2021), Békés et al. (2022), Mei et al., 2022)
Pathways Affected by Circadian Rhythms	Autophagy Pathway: BMAL1 regulates autophagy and limits disc cell senescence	Maiese (2017), Mei et al. (2022)
	Inflammation Pathway: Inflammation Pathway: Circadian control of NF-κB and IL-6/TNF-α modulates inflammation in disc tissue	Baxter and Ray (2020), Zhang et al. (2022b)
	Metabolic Pathways (AMPK/mTOR): Controls energy balance and nutrient sensing in disc cells	Lai et al. (2023), Zhang et al. (2019), Chappuis et al. (2013)
	Oxidative Stress Response: Circadian genes support antioxidant defense in disc cells	Lee et al. (2021b), Jordan and Lamia (2013)
Potential Therapeutic Targets	BMAL1 Activation: Enhances autophagy, reduces inflammation, and slows degeneration	Maiese (2017) Xing et al. (2021)
	REV-ERBα Modulation: Targets REV-ERBα to reduce inflammation and stabilize the matrix	Yu et al. (2021), Mei et al. (2022), Békés et al. (2022)
	AMPK Activation: Enhances energy balance and autophagy, preserving disc cell function	Lai et al. (2023), Zhang et al. (2019)
	SIRT1 Activation: Boosts autophagy and reduces oxidative stress, protecting disc cells	Ma et al. (2022), Xing et al. (2021)
Potential Therapeutic Drugs	REV-ERBα Agonists: Regulate BMAL1 expression and inflammatory responses, with improved specificity and bioavailability	Yu et al. (2021), Békés et al. (2022)
	SIRT1 Activators: Promote autophagy and reduce oxidative damage, targeting disc cells	Guo et al. (2023), Xing et al. (2021)
	AMPK Activators: Enhance energy homeostasis and autophagy, slowing disc degeneration	Lai et al. (2023), Zhang et al. (2019)
	Corticosteroids (Timed): Administered with circadian rhythms to reduce inflammatory cytokines and protect the disc matrix	Baxter and Ray (2020), Lee et al. (2021b), Zhang et al. (2022b)

BMAL1, Brain and Muscle ARNT-Like 1; CLOCK, circadian locomotor output cycles kaput; PER1/PER2, Period Circadian Regulator 1/2; REV-ERBα, Nuclear Receptor Subfamily 1 Group D Member 1 (also known as NR1D1); NF-κB, Nuclear Factor kappa-light-chain-enhancer of activated B cells; IL-6, Interleukin 6; TNF-α, tumor necrosis factor alpha; AMPK, AMP-Activated Protein Kinase; mTOR, mechanistic target of rapamycin; SIRT1, Sirtuin 1; HIF1α, Hypoxia-Inducible Factor 1-alpha.

Although studies on circadian rhythms in the intervertebral disc remain limited, current evidence suggests that disc cells also possess intrinsic circadian characteristics. Expression of CLOCK, BMAL1, PER, and CRY in disc tissue changes with circadian cycles and may regulate matrix metabolism and cell survival, thereby contributing to disc homeostasis (Schmitt et al., 2018; Li B. et al., 2023). For example, BMAL1 is expressed in disc-related cells and is involved in matrix regulation through effects on MMP (matrix metalloproteinase) and ADAMTS (a disintegrin and metalloproteinase with thrombospondin motifs) family genes (Chen et al., 2023). CLOCK expression is associated with disc cell proliferation and autophagy, and CLOCK dysfunction can reduce autophagy in nucleus pulposus cells and further accelerate IDD. BMAL1 deficiency can also increase expression of inflammatory mediators such as TNF-α and IL-1β, activate the NF-κB pathway, and aggravate disc degeneration, suggesting that circadian regulation in the intervertebral disc may influence tissue health partly through control of inflammatory signaling (Dudek et al., 2023).

## 3 Circadian rhythm disruption and IDD

**FIGURE 2:**
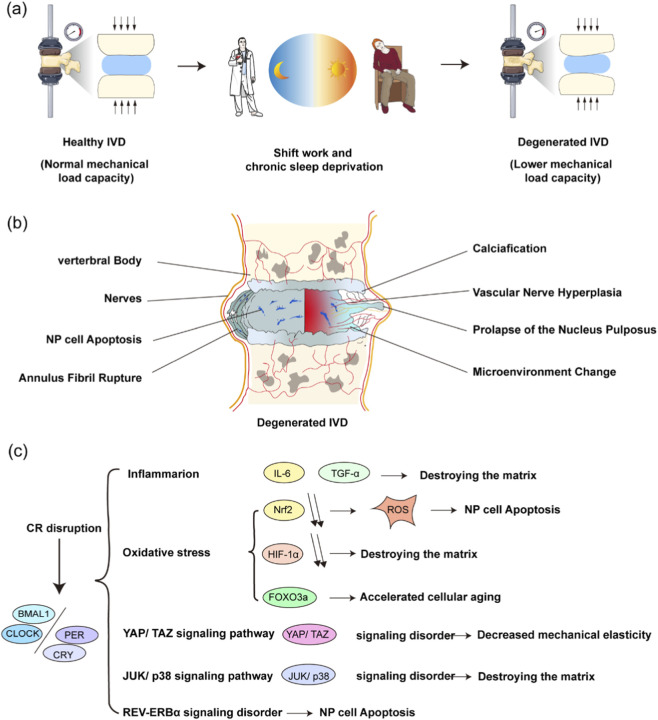
The contribution of circadian rhythm disruption to disc degeneration. **(a)** Disordered sleep-wake patterns caused by shift work or insomnia may promote disc degeneration, including disc deformation and abnormal mechanical loading. **(b)** Degeneration-related changes in the disc microenvironment may lead to nucleus pulposus (NP) cell apoptosis, annulus fibrosus (AF) rupture, and loss of NP integrity. **(c)** Circadian disruption alters clock gene expression and affects pathways involving Nrf2, HIF-1α, FOXO3a, YAP/TAZ, and JNK/p38, while increasing inflammatory mediators such as IL-6 and TNF-α. These changes contribute to matrix breakdown, NP cell apoptosis, and reduced mechanical elasticity.

Circadian rhythm disruption caused by shift work, recurrent jet lag, and sleep deprivation has increasingly been recognized as an important factor affecting disc health in recent years (Ding et al., 2021) (Figure 2a) Studies have shown that shift workers often develop marked circadian disturbance because of long-term exposure to artificial light and irregular sleep-wake schedules, resulting in abnormal expression of core clock genes such as CLOCK and BMAL1. This disturbance not only promotes disc matrix degradation through regulation of inflammatory genes, including IL-6 and TNF-α, but also interferes with cellular metabolism and impairs the proliferative and reparative capacity of disc cells (Dudek et al., 2017). A recent study further showed that shift workers exhibit reduced activity of the Nrf2 signaling pathway, which is important in the response to oxidative stress. Suppression of Nrf2 increases intracellular oxidative stress, induces early apoptosis of disc cells, and accelerates IDD progression (Kang et al., 2020).

Frequent jet lag and chronic sleep loss are also important contributors to circadian disruption. These conditions can aggravate dysregulation of oxygen homeostasis in disc cells by affecting stabilization of HIF-1α (hypoxia-inducible factor-1α). Under normal conditions, HIF-1α helps protect disc cells by supporting their adaptation to the hypoxic environment. However, disruption of circadian rhythms suppresses HIF-1α expression, leading to reduced matrix synthesis, impaired autophagy, and ultimately faster progression of IDD (Suyama et al., 2016; Li et al., 2021). In addition, recent work has linked abnormal expression of PER1 and PER2 to IDD under circadian disruption. Dysregulation of these genes not only disturbs the metabolic rhythm of disc cells, but also promotes cellular senescence and matrix degradation through interference with FOXO3a-dependent antioxidant responses (Zhou et al., 2019).

Mechanical stress is a well-recognized factor in intervertebral disc degeneration, and recent studies suggest that circadian rhythms are important in shaping how disc cells respond to mechanical loading. Circadian genes appear to influence time-dependent responses to mechanical stress through regulation of the YAP/TAZ pathway (Zheng-Wei et al., 2023). During the daytime, YAP/TAZ activity is increased, which promotes matrix protein synthesis and cell proliferation and helps maintain disc elasticity under loading. At night, YAP/TAZ activity declines, allowing cells to enter a reparative state and reduce the cumulative mechanical injury generated during the day (Hu et al., 2024). Recent studies indicate that circadian disruption disturbs this finely balanced system. It has been reported that circadian rhythm disruption causes abnormal activation of the JNK and p38 MAPK pathways, both of which are important in stress responses induced by mechanical loading. Enhanced nocturnal activation of these pathways promotes apoptosis and matrix breakdown, thereby accelerating IDD (Cui et al., 2021) (Figure 2b). Further work has highlighted the role of TGF-β signaling in the interaction between circadian rhythms and mechanical stress. Under normal conditions, TGF-β protects the disc by promoting matrix synthesis and extracellular matrix repair, whereas circadian disturbance reduces the effectiveness of this pathway, weakens resistance to mechanical stress, and increases susceptibility to IDD (Morris et al., 2021).

In recent years, animal studies have provided additional insight into how circadian rhythm disruption contributes to IDD. Knockout and mutant models have made it possible to define the role of circadian genes in disc homeostasis more clearly. For example, BMAL1 knockout mice show obvious features of IDD, underscoring the key role of BMAL1 in circadian regulation and matrix metabolism (Wang et al., 2022). Likewise, deletion of REV-ERBα can trigger IDD, further supporting the importance of circadian regulation through autophagic and metabolic pathways (Zhou et al., 2024). Experimental disruption of circadian rhythms in animals has also yielded important findings. For instance, alternating light-dark cycles have been used to mimic the effects of shift work or recurrent jet lag, and these models show significantly aggravated disc degeneration. In such models, SIRT1 levels are markedly reduced, leading to impaired autophagy and disturbed cellular energy metabolism, which ultimately accelerates IDD (Hao et al., 2022). Together, these studies reveal the multiple ways in which circadian rhythm disruption contributes to IDD and provide a theoretical basis for the future development of circadian-based therapeutic strategies (Figure 2c).

## 4 Crosstalk between IDD and circadian rhythms

**FIGURE 3:**
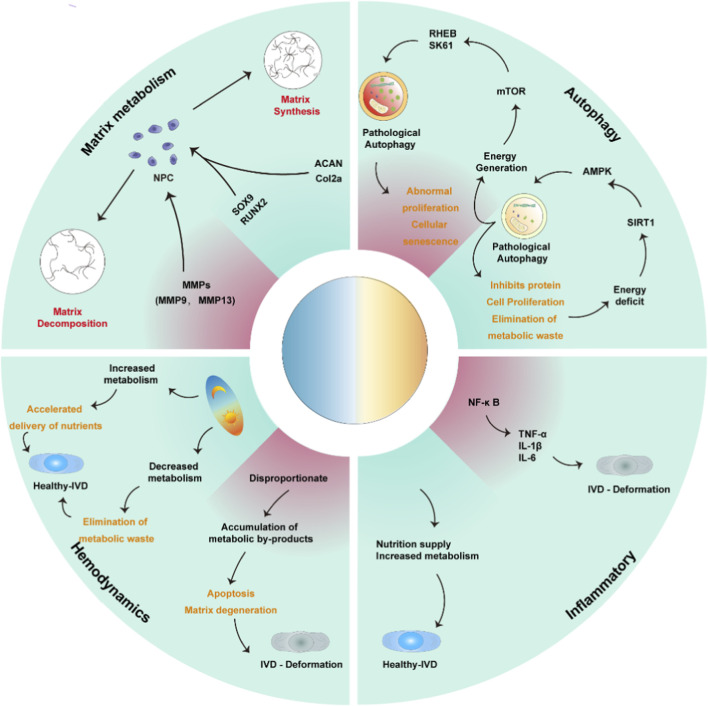
Under stable circadian conditions, osteogenesis-related genes such as SOX9 and RUNX2, together with matrix synthesis-related genes such as ACAN and Col2a, help maintain extracellular matrix production. In contrast, matrix-degrading enzymes, including MMP9 and MMP13, promote matrix breakdown and accelerate IDD. A stable circadian rhythm is also essential for active cellular and nutrient metabolism. Under energy-deficient conditions, physiological autophagy is induced through the SIRT1/AMPK pathway to support normal metabolic needs. When circadian rhythms are disrupted, however, pathological autophagy may be activated through mTOR/RHEB, while the NF-κB inflammatory pathway is also stimulated. This leads to accumulation of inflammatory mediators such as TNF-α, IL-1β, and IL-6, resulting in abnormal cell proliferation, buildup of metabolic by-products, cellular senescence, cell death, and eventually IDD.

The intervertebral disc is mainly composed of the nucleus pulposus, annulus fibrosus, and cartilaginous endplates, and these structures are all essential for maintaining disc integrity and function. CLOCK, BMAL1, PER, and CRY are expressed in the different cell types that make up these structures, and their rhythmic expression is closely associated with cyclic regulation of matrix metabolism, autophagy, inflammatory responses, and nutrient transport. Together, these processes are important for preserving disc homeostasis (Figure 3).

### 4.1 Matrix metabolism

Matrix components such as collagen and proteoglycans are essential for maintaining the structure and function of the intervertebral disc. Their synthesis and degradation are tightly regulated by circadian rhythms. The CLOCK/BMAL1 heterodimer coordinates matrix synthesis during the circadian cycle by regulating genes such as COL2A1 and ACAN (Sherratt et al., 2019). For example, BMAL1 has been shown to directly regulate the expression of aggrecan and collagen II in disc-related cells (Morris et al., 2021). Matrix synthesis is generally enhanced during the daytime, when mechanical loading increases, and this process depends in part on BMAL1-mediated activation of protein synthesis through the mTOR pathway, which helps maintain disc elasticity. At night, when mechanical loading decreases, circadian genes regulate matrix-degrading enzymes to promote matrix turnover and remodeling under lower mechanical stress (Ding et al., 2021). In annulus fibrosus cells, expression of CRY1 and CLOCK is closely related to cell proliferation and matrix metabolism. CRY1 can indirectly regulate MMP expression by inhibiting CLOCK/BMAL1 activity, thereby affecting matrix degradation in the annulus fibrosus (Patke et al., 2017; Yoshida et al., 2014). This mechanism is important for maintaining annulus fibrosus stability under daytime mechanical loading (Ray et al., 2020). In cartilage endplate cells, circadian gene expression is also closely related to bone metabolism and matrix synthesis. BMAL1 expression in the cartilage endplate is positively associated with osteogenic and chondrogenic genes such as RUNX2 and SOX9, suggesting that BMAL1 contributes to maintenance of both the disc and adjacent bone tissue through regulation of these genes (Delisle et al., 2021).

When circadian rhythms are disrupted, however, the normal rhythm of matrix synthesis may be disturbed, leading to accelerated matrix breakdown and disc degeneration. MMPs and their tissue inhibitors (TIMPs) are major regulators of disc matrix metabolism (Roberts et al., 2006). CLOCK/BMAL1 has been shown to control matrix degradation and remodeling during the circadian cycle through regulation of MMP-3 and MMP-13 expression (He et al., 2018). Under physiological conditions, MMP expression increases at night and contributes to matrix turnover, which is consistent with nocturnal repair and remodeling activity (Ding et al., 2021; Morris et al., 2021). In contrast, circadian disruption reduces disc tolerance to abnormal mechanical loading and increases expression of matrix-degrading enzymes such as MMP-3 and MMP-13, thereby accelerating matrix damage and annulus fibrosus injury (Albazal et al., 2021).

These findings highlight the importance of circadian regulation in maintaining disc structure and function through control of matrix metabolism.

### 4.2 Autophagy

Autophagy is the process by which cells remove damaged organelles and metabolic waste through the action of autophagy-related genes. It can be broadly divided into physiological autophagy and stress-induced autophagy (Barbosa et al., 2018). Almost all stressors that disturb cellular homeostasis can trigger autophagy. Physiological autophagy serves as a protective mechanism and plays an important role in maintaining cellular homeostasis and biosynthetic function (Chen et al., 2024). In contrast, stress-induced autophagy is more closely linked to aging, immune disorders, and neurodegenerative diseases (Klionsky et al., 2021). Current evidence suggests that disc autophagy is closely associated with circadian regulation and that formation of autophagic vacuoles is influenced by clock genes (Rabi et al., 2019; Ma et al., 2011). Clock genes have been shown to regulate autophagosome formation and degradation through control of autophagy-related genes, thereby enhancing nocturnal autophagic activity and facilitating waste clearance and delayed cellular aging (Oyama et al., 2021). Conversely, circadian disruption or PER2 mutation can impair autophagy and promote accumulation of metabolic waste, which may further aggravate ID (Zhang J. et al., 2022). These findings suggest that autophagy dysregulation, which is influenced by clock genes, is closely associated with nucleus pulposus degeneration and aging.

AMPK (adenosine monophosphate-activated protein kinase) and mTOR (mechanistic target of rapamycin) are two major pathways involved in autophagy regulation, and both are closely controlled by circadian rhythms. CLOCK and BMAL1 affect energy metabolism and cell survival in disc cells through genes associated with the AMPK and mTOR pathways (Li Z. et al., 2023). As a cellular energy sensor, AMPK is activated under energy-deficient conditions, where it promotes energy production and autophagy while suppressing protein synthesis. Circadian rhythms influence AMPK activity through regulation of SIRT1, thereby shaping metabolic rhythms in cells (Lai et al., 2023). BMAL1 has been reported to affect AMPK activity indirectly through SIRT1 expression, reducing energy expenditure and promoting autophagy during the night phase (Ma et al., 2022). By contrast, the mTOR pathway is activated under nutrient-rich and energy-sufficient conditions to promote protein synthesis and cell proliferation. CLOCK/BMAL1 regulates downstream targets of the mTOR pathway, such as RHEB and S6K1, thereby influencing disc cell proliferation and metabolic activity across the circadian cycle (Li et al., 2024). Disruption of circadian rhythms may cause overactivation of mTOR, leading to abnormal proliferation and premature senescence of disc cells. For example, BMAL1 in nucleus pulposus cells affects cell proliferation and metabolism through the mTOR pathway (Cao, 2018). Loss of BMAL1 can result in hyperactivation of mTOR, which drives uncontrolled proliferation and accelerates cellular senescence (Engin, 2017). Together, these findings suggest that circadian regulation of autophagy may help protect the disc from degeneration.

### 4.3 Inflammatory

Circadian genes are closely linked to inflammatory pathways, particularly in intervertebral disc cells, where they modulate the timing and magnitude of inflammatory responses through regulation of inflammation-related genes. BMAL1, for example, plays an important role in suppressing NF-κB signaling (Dudek et al., 2017). NF-κB is a key transcription factor involved in expression of inflammatory mediators such as TNF-α and IL-1β, both of which contribute to IDD (Zhang et al., 2021; Dong et al., 2019). Under normal circadian conditions, BMAL1 directly or indirectly limits NF-κB activity and thereby reduces inflammatory mediator production. When circadian rhythms are disrupted, such as under sleep deprivation or clock gene mutation, this inhibitory effect is weakened, leading to a stronger inflammatory response, increased matrix degradation, and faster disc degeneration. In addition, CLOCK also participates in inflammatory regulation within the disc through effects on IL-6 and IL-1β expression (Dudek et al., 2017). Abnormal CLOCK activity can therefore lead to dysregulated release of inflammatory mediators and aggravate disc inflammation and degeneration.

The strength and duration of inflammatory responses are clearly influenced by circadian timing (Man et al., 2021). Studies have shown that inflammation in intervertebral disc tissue follows an obvious circadian pattern, with inflammatory mediator expression and release often peaking at night in parallel with systemic immune rhythms (Peng et al., 2022). Disturbance of circadian rhythms or mutation of clock genes can disrupt this temporal control and lead to inappropriate overexpression of inflammatory factors. For example, mice with circadian gene mutations show stronger inflammatory responses and more severe IDD in experimental models (Suyama et al., 2016; Morris et al., 2021). These findings emphasize that intact circadian regulation is important for controlling inflammation in the disc and slowing degenerative progression.

### 4.4 Hemodynamics

As an avascular tissue, the intervertebral disc depends mainly on diffusion and osmotic processes for nutrient supply. Studies suggest that circadian rhythms influence nutrient delivery and waste clearance around the disc by regulating blood flow, osmotic pressure, and local cellular metabolic activity (Morris et al., 2021). Circadian activity in nucleus pulposus and annulus fibrosus cells affects diffusion and distribution of oxygen, glucose, and amino acids within the matrix. During the day, cellular activity is higher and metabolic demand increases, which is accompanied by greater demand for oxygen and nutrient supply within the disc. At night, metabolic demand decreases and changes in osmotic pressure help facilitate removal of waste products (Zhang et al., 2020). Circadian disruption may disturb this balance and contribute to IDD by impairing nutrient delivery. For example, chronic circadian disturbance can reduce oxygen and nutrient availability within the disc, leading to accumulation of metabolic byproducts, cell death, and matrix degradation. It has also been reported that circadian disruption alters the acidic environment of the matrix, further worsening nutrient deficiency and degeneration. Under acidic conditions, disc cells are less able to survive, matrix synthesis declines, and matrix breakdown is enhanced, all of which accelerate degeneration (Ding et al., 2021).

In summary, comparison with the role of circadian rhythms in tissues such as muscle and bone provides useful clues for understanding their unique functions in disc biology. Circadian rhythms may help preserve disc health by regulating matrix turnover, cell proliferation, autophagy, nutrient supply, and waste clearance. In addition, interactions between circadian genes and factors such as mechanical loading and inflammation may play an important role in the onset and progression of IDD. These cross-disciplinary perspectives not only improve our understanding of disc homeostasis, but also offer potential directions for the development of circadian-based therapies. Such strategies may include circadian modulators, gene-based approaches, or related interventions aimed at slowing disc degeneration and improving patient outcomes.

## 5 Circadian regulation as a potential therapeutic strategy

Phototherapy is a non-invasive approach that helps restore normal circadian rhythms by adjusting the light-dark cycle, and it has been widely used in circadian-related disorders. Its main effect depends on activation of photosensitive pathways in the suprachiasmatic nucleus (SCN), which then regulate the expression of core clock genes such as PER1, CRY1, and BMAL1 (Lee J. et al., 2021). By correcting clock gene expression, phototherapy helps re-establish normal circadian periodicity and thereby influences downstream processes, including inflammation and cellular metabolism (Touitou et al., 2016). Studies have shown that light therapy can significantly reduce inflammatory mediators in the disc, such as IL-6 and TNF-α, both of which are important in IDD (Dibner et al., 2010). In addition, phototherapy may slow degeneration by regulating stress-related pathways such as JNK and p38 MAPK, thereby reducing mechanical stress-induced injury in disc cells (Sutton et al., 2018). In high-risk populations, especially shift workers and individuals with circadian disruption, light therapy may help restore biological rhythms and lower the risk of IDD. Timed feeding is another intervention that aligns meal timing with circadian rhythms in order to optimize metabolism and reduce cellular stress, thereby protecting the disc from degeneration. Studies have shown that timed feeding can maintain cellular energy balance by regulating the AMPK and mTOR pathways (Hu et al., 2019). These pathways are closely associated with circadian rhythms and contribute to limiting cellular injury induced by metabolic imbalance (Acosta-Rodríguez et al., 2022). In disc cells, timed feeding promotes autophagy and reduces oxidative stress by increasing the expression of SIRT1 and PGC-1α, thereby delaying cellular senescence and matrix breakdown (Ma et al., 2022). Timed feeding may also enhance adaptation to hypoxia through upregulation of HIF-1α, thereby helping to maintain the structural and functional integrity of the intervertebral disc (Zhang et al., 2019). Further studies suggest that timed feeding not only affects gene expression but also regulates the biological activity of disc cells through protein phosphorylation. For example, timed feeding can reduce mTORC1 activity, thereby lowering the burden of protein synthesis and decreasing stress caused by protein aggregation (Lu et al., 2020). This effect may be particularly relevant for slowing IDD under conditions of high metabolic demand.

Chronotherapy is a circadian-based treatment strategy in which drugs are given at specific biological phases to improve efficacy and reduce adverse effects (Lee et al., 2021b). For example, nighttime administration of anti-inflammatory drugs has been shown to more effectively inhibit NF-κB signaling, reduce release of inflammatory mediators, and slow IDD progression. This time-dependent effect highlights the importance of circadian rhythms in determining drug response (Baxter and Ray, 2020). Additional studies indicate that chronotherapy can also enhance matrix protein synthesis by giving treatment during periods of high metabolic activity, especially for key genes such as COL2A1 and ACAN (Zhang et al., 2022b). This strategy may improve matrix synthesis efficiency while reducing off-target effects during less responsive periods, thereby increasing tolerance and treatment adherence. Circadian regulation of drug action has therefore become an important area in attempts to delay IDD. For instance, REV-ERBα agonists have been reported to suppress inflammation- and matrix degradation-related genes by modulating nocturnal BMAL1 expression (Yu et al., 2021). This not only reduces matrix breakdown but may also help preserve disc structure by supporting matrix synthesis. In addition, SIRT1 agonists given at different circadian phases can markedly affect autophagy in disc cells. Nighttime administration has been shown to increase the expression of autophagy-related genes such as LC3 and ATG7, promote autophagosome formation, and facilitate clearance of intracellular metabolic waste, thereby delaying disc cell senescence (Maiese, 2017). Circadian regulation of drug effects may also involve protein ubiquitination and degradation. For example, PROTAC technology (PROteolysis TArgeting Chimeras) has been explored to regulate degradation of specific proteins in a circadian-dependent manner. This approach may selectively eliminate abnormally expressed matrix-degrading enzymes in disc cells and thereby reduce excessive matrix destruction (Békés et al., 2022).

Maintaining a regular sleep pattern is also important for preventing IDD. Studies have shown that stable sleep-wake schedules help synchronize CLOCK and BMAL1 expression, preserve normal cellular metabolic rhythms, and reduce matrix degradation and apoptosis (Chen et al., 2023). In high-risk groups, particularly night shift workers, restoring circadian rhythm through adjustment of sleep schedules may significantly reduce the risk of IDD(47). In addition, healthy sleep habits may lessen mechanically induced disc injury by suppressing abnormal activation of the p38 MAPK and JNK pathways (Mei et al., 2021). This protective effect of sleep regulation may be relevant not only to IDD but also to other degenerative conditions. Daily behavioral regulation, including proper light exposure, regular meal timing, and moderate exercise, is also an important way to maintain circadian rhythms. Studies suggest that sufficient sunlight exposure can promote matrix synthesis and repair through regulation of PER2 expression. Regular feeding and moderate exercise can also improve cellular energy metabolism and antioxidant capacity through the AMPK and SIRT1 pathways, thereby supporting disc health (Chappuis et al., 2013). Behavioral adjustment further includes improving sleep environment and sleep duration. Proper sleep has been shown to reduce cortisol (CORT) release, which may lessen stress-related loading on the disc (Xing et al., 2021). In addition, moderate exercise can promote matrix synthesis and cellular repair by influencing IGF-1 and TGF-β signaling (Wang et al., 2021).

Based on current studies, a new hypothesis can be proposed: circadian regulation may offer new therapeutic opportunities for IDD by influencing autophagy and matrix metabolism in disc cells. In particular, changes in clock gene expression, such as BMAL1 and PER2, may enhance autophagy through regulation of genes such as LC3 and ATG5, thereby reducing metabolic waste accumulation and delaying IDD progression (Peek et al., 2017). This hypothesis is supported not only by existing molecular studies but also by observations that circadian regulation protects tissue homeostasis in other organ systems. These findings suggest that modulation of circadian rhythms may be explored as a new treatment strategy for IDD.

The clinical potential of circadian regulation is considerable. First, non-pharmacological interventions such as phototherapy and timed feeding may help restore circadian alignment and thereby slow IDD progression. This may be especially useful in early intervention, particularly in high-risk individuals who have not yet developed clear symptoms. Second, optimizing drug timing through chronotherapy may improve treatment efficacy while reducing side effects, which is particularly relevant for patients requiring long-term therapy. Future research should focus on clinical trials to evaluate the effectiveness of circadian-based interventions in the prevention and treatment of IDD and to assess the feasibility of personalized therapeutic strategies. For example, trials could compare the effects of administering the same treatment at different times of day in order to determine the influence of circadian timing on IDD outcomes. Combined strategies involving phototherapy, timed feeding, and pharmacological treatment may also be explored to maximize therapeutic benefit. In addition, circadian regulation may serve as a preventive approach to reduce the incidence of IDD. In younger high-risk populations in particular, early circadian intervention may delay or prevent disease onset, improve quality of life, and reduce future healthcare burden.

## 6 Directions for future research

Multi-omics approaches integrating genomics, proteomics, and metabolomics provide a systematic way to investigate how circadian rhythms affect intervertebral disc health. At the genomic level, studies have shown that CLOCK and BMAL1 regulate downstream genes such as PER1 and PER2, which directly influence metabolic rhythms and stress responses in disc cells. Altered expression or mutation of these core clock genes may disturb circadian regulation and thereby accelerate IDD progression (Pan et al., 2020; Zhang and Kay, 2010).

Proteomic studies have further identified post-translational regulatory events closely linked to circadian rhythms. For example, phosphorylation of PER2 is an important step in circadian regulation and is controlled by CKIε (casein kinase 1 ε), while its degradation depends on the ubiquitin-proteasome system (Meng et al., 2008). In addition, REV-ERBα participates in lipid metabolism and autophagy through a negative feedback relationship with core clock genes, and this process is important for maintaining metabolic homeostasis in disc cells (Solt et al., 2012).

Metabolomic evidence has also shown that many metabolites fluctuate rhythmically under circadian control. For instance, the activity of the AMPK and mTOR pathways in intervertebral disc cells changes across the circadian cycle, directly affecting energy metabolism and autophagy (Guo et al., 2024). By combining genomic, proteomic, and metabolomic data, it becomes possible to more comprehensively understand how circadian rhythms influence disc health through coordinated effects on gene expression, protein modification, and metabolic pathways.

These multi-omics technologies are also important for identifying new biomarkers related to circadian regulation that may be useful for early diagnosis and personalized treatment of IDD. For example, proteomic studies have identified circadian variation in NRF2 (Nuclear Factor erythroid 2-Related Factor 2), which is associated with antioxidant responses, and activation of NRF2 appears to help protect disc cells from oxidative stress (Kang et al., 2020). Metabolomics studies have also identified circadian changes in SIRT1-related metabolites, such as NAD+, which may serve as early warning markers for predicting IDD progression (Nakahata et al., 2008). In addition, such biomarkers may help clarify how circadian disruption specifically affects disc health and may support the development of individualized therapies. For example, circadian disturbance may increase release of inflammatory mediators through the NF-κB pathway, while time-dependent changes in markers such as IL-6 and TNF-α may provide useful targets for optimizing chronotherapy (Wang et al., 2018).

Individual differences in circadian rhythms remain an important challenge in clinical research. Circadian characteristics can vary widely among individuals because of CLOCK polymorphisms, PER2 mutations, lifestyle factors, and environmental influences such as light exposure and sleep habits (Guo et al., 2023). These differences may not only affect the impact of circadian rhythms on disc health, but may also influence the timing of drug metabolism and treatment response. Genomic analysis may help identify polymorphisms in CLOCK and BMAL1 and thereby define inter-individual variation in circadian traits. Integration of metabolomics data may further clarify how AMPK and mTOR activity fluctuates at different times of day, which could support the design of more personalized time-based treatments (Jordan and Lamia, 2013).

Incorporating circadian factors into IDD clinical trial design is an important direction for future research. For example, nighttime administration of a SIRT1 agonist has been reported to enhance autophagy in disc cells by upregulating genes such as LC3 and ATG7, thereby promoting intracellular waste clearance and slowing IDD progression (Mei et al., 2022). In clinical trials, time-based dosing strategies may therefore be needed to improve efficacy while reducing adverse effects. At the same time, applying circadian regulation in clinical research also presents several challenges, including the need for accurate monitoring of circadian status and the need to integrate information on individual genetic variation when designing optimal dosing schedules. In addition, reliable biomarkers, such as circadian variation in REV-ERBα expression, still need to be developed for evaluating time-dependent therapeutic effects (Shi et al., 2022).

Although the influence of circadian rhythms on disc health has been increasingly studied, other physiological rhythms may also be relevant. Seasonal variation, for example, has been reported to affect vitamin D synthesis and thyroid hormone levels, both of which may influence disc health by regulating matrix metabolism and cell proliferation (Brayda-Bruno et al., 2017). Seasonal changes may also alter inflammatory responses in the intervertebral disc by affecting the expression of mediators such as TNF-α and IL-1β. These effects may contribute to seasonal patterns in IDD, particularly in individuals with impaired immune function (Wang et al., 2020). Beyond circadian and seasonal rhythms, other biological rhythms, including lunar and annual rhythms, may also affect disc health. Some studies suggest that lunar rhythms can influence melatonin secretion, which in turn may affect P53-mediated cell-cycle regulation and apoptosis, both of which are important for disc cell survival (Wang et al., 2022). Annual rhythms have also been linked to variation in sex hormone levels, which may influence disc matrix metabolism and repair through regulation of ERα and AR receptors (Wang et al., 2020). Future work should therefore examine circadian rhythms together with other physiological rhythms in more integrated studies. Such approaches may provide a more complete understanding of disc biology and may help clarify how different rhythms interact to affect disc health. For example, multi-omics integration could help identify how pathways such as Wnt signaling vary across different rhythmic states and how these changes influence matrix synthesis and cell proliferation (Lai et al., 2023).

## 7 Conclusion

Circadian rhythms play an important role in maintaining intervertebral disc health. By regulating cellular metabolism, matrix synthesis, and inflammatory activity, circadian rhythms help preserve normal disc function and structural integrity. Core clock genes, including CLOCK, BMAL1, and PER2, are involved in these processes through regulation of autophagy, oxidative stress, and metabolic pathways, thereby supporting disc cell survival and matrix stability. When circadian rhythms are disrupted, IDD progression may accelerate and inflammatory mediator release may increase, further damaging disc tissue.

Circadian-based regulation also has considerable clinical potential for the prevention and treatment of IDD. Interventions such as chronotherapy, phototherapy, and timed feeding may help restore or optimize circadian rhythms, and therefore may slow disc degeneration and improve patient outcomes. Although current results are encouraging, the clinical feasibility and true therapeutic value of these strategies still need to be confirmed in large-scale and long-term clinical studies.

Future research should continue to investigate the role of circadian rhythms in disc biology, especially through interdisciplinary studies combining mechanistic work, multi-omics analysis, and clinical research. Such efforts may support the development of more precise and individualized treatment strategies and provide new ideas for the prevention and treatment of IDD. The relationship between circadian rhythms and intervertebral disc health remains an area worthy of continued and in-depth investigation.

## Glossary

IDD: intervertebral disc degeneration
NP: nucleus pulposus
AF: annulus fibrosus
CEPs: cartilaginous endplates
ECM: extracellular matrix
SCN: suprachiasmatic nucleus
CLOCK: Circadian Locomotor Output Cycles Kaput
BMAL1: brain and muscle ARNT-like 1
PER: period
CRY: cryptochrome
TTFL: transcription-translation feedback loop
ASD: adjacent segment disease
CK1ε/δ: casein kinase 1 ε/δ
FBXL3: F-box and leucine-rich repeat protein 3
FBXL21: F-box and leucine-rich repeat protein 21
NLS: nuclear localization signal
NES: nuclear export signal
mTORC1: mechanistic target of rapamycin complex 1
Pparα: peroxisome proliferator-activated receptor alpha
Srebp1: sterol regulatory element-binding protein 1
G6pc: glucose-6-phosphatase catalytic subunit
Pepck: phosphoenolpyruvate carboxykinase
PGC-1α: peroxisome proliferator-activated receptor gamma coactivator 1-alpha
RUNX2: runt-related transcription factor 2
OPG: osteoprotegerin
MMP: matrix metalloproteinase
ADAMTS: a disintegrin and metalloproteinase with thrombospondin motifs
TNF-α: tumor necrosis factor-alpha
IL: interleukin
Nrf2: nuclear factor erythroid 2-related factor 2
HIF-1α: hypoxia-inducible factor-1 alpha
JNK: c-Jun N-terminal kinase
MAPK: mitogen-activated protein kinase
TGF-β: transforming growth factor-beta
SIRT1: sirtuin 1
TIMP: tissue inhibitor of metalloproteinase
AMPK: adenosine monophosphate-activated protein kinase
mTOR: mechanistic target of rapamycin
RHEB: Ras homolog enriched in brain
NF-κB: nuclear factor kappa-B
PROTAC: proteolysis-targeting chimera
LC3: microtubule-associated protein 1 light chain 3
ATG: autophagy-related gene
CORT: cortisol
IGF-1: insulin-like growth factor 1
CKIε: casein kinase 1 epsilon
NAD+: nicotinamide adenine dinucleotide
ERα: estrogen receptor alpha
AR: androgen receptor
